# *BaiCD* gene cluster abundance is negatively correlated with *Clostridium difficile* infection

**DOI:** 10.1371/journal.pone.0196977

**Published:** 2018-05-08

**Authors:** Philipp Solbach, Patrick Chhatwal, Sabrina Woltemate, Evelina Tacconelli, Michael Buhl, Markus Gerhard, Christoph K. Thoeringer, Maria J. G. T. Vehreschild, Nathalie Jazmati, Jan Rupp, Michael P. Manns, Oliver Bachmann, Sebastian Suerbaum

**Affiliations:** 1 Hannover Medical School, Institute of Medical Microbiology and Hospital Epidemiology, Hannover, Germany; 2 Hannover Medical School, Department of Gastroenterology, Hepatology and Endocrinology, Hannover, Germany; 3 German Center for Infection Research (DZIF), partner site Hannover-Braunschweig, Hannover-Braunschweig, Germany; 4 Tübingen University Hospital, Division of Infectious Diseases, Department of Internal Medicine 1, Tübingen, Germany; 5 German Center for Infection Research (DZIF), partner site Tübingen, Tübingen, Germany; 6 Tübingen University Hospital, Institute of Medical Microbiology and Hygiene, Tübingen, Germany; 7 Technische Universität München, Institute for Medical Microbiology, Immunology and Hygiene, Munich, Germany; 8 German Center for Infection Research (DZIF), partner site Munich, Munich, Germany; 9 Technische Universität München, Department of Internal Medicine II, Klinikum rechts der Isar, Munich, Germany; 10 University Hospital of Cologne, 1st Department of Internal Medicine, Cologne, Germany; 11 German Center for Infection Research (DZIF), partner site Bonn-Cologne, Bonn-Cologne, Germany; 12 University of Cologne, Institute for Medical Microbiology, Immunology and Hygiene, Cologne, Germany; 13 University Hospital Schleswig-Holstein, Department of Infectious Diseases and Microbiology, Lübeck, Germany; 14 German Center for Infection Research (DZIF), partner site Hamburg-Borstel-Lübeck, Hamburg-Borstel-Lübeck, Germany; 15 LMU Munich, Max von Pettenkofer Institute, München, Germany; Universidad Andres Bello, CHILE

## Abstract

**Background:**

*Clostridium difficile* infection (CDI) is a major cause of hospital-acquired diarrhea. Secondary bile acids were shown to confer resistance to colonization by *C*. *difficile*. 7α-dehydroxylation is a key step in transformation of primary to secondary bile acids and required genes have been located in a single bile acid-inducible (bai) operon in *C*. *scindens* as well as in *C*. *hiranonis*, two *Clostridium* sp. recently reported to protect against *C*. *difficile* colonization.

**Aim:**

To analyze *baiCD* gene abundance in *C*. *difficile* positive and negative fecal samples.

**Material & methods:**

A species-specific qPCR for detecting *baiCD* genes was established. Fecal samples of patients with CDI, asymptomatic toxigenic *C*. *difficile* colonization (TCD), non-toxigenic *C*. *difficile* colonization (NTCD), of *C*. *difficile* negative (NC) patients, and of two patients before and after fecal microbiota transplantation (FMT) for recurrent CDI (rCDI) were tested for the presence of the *baiCD* genes.

**Results:**

The prevalence of the *baiCD* gene cluster was significantly higher in *C*. *difficile* negative fecal samples than in samples of patients diagnosed with CDI (72.5% (100/138) vs. 35.9% (23/64; p<0.0001). No differences in *baiCD* gene cluster prevalence were seen between NC and NTCD or NC and TCD samples. Both rCDI patients were *baiCD*-negative at baseline, but one of the two patients turned positive after successful FMT from a *baiCD*-positive donor.

**Conclusion:**

Fecal samples of CDI patients are less frequently *baiCD*-positive than samples from asymptomatic carriers or *C*. *difficile*-negative individuals. Furthermore, we present a case of *baiCD* positivity observed after successful FMT for rCDI.

## Introduction

*Clostridium difficile* infection (CDI) is the major cause of hospital-acquired diarrhea and is associated with significant morbidity and mortality [1–3]. Broad spectrum antibiotic treatment disrupts the commensal host microbiota, thereby increasing the risk for CDI. A crucial pathophysiologic concept in this context is a breakdown of colonization resistance [4–6], which involves direct and indirect mechanisms. Direct mechanisms include the competition for nutrients, the conversion of nutrients (e.g. *small chain fatty acids*, SCFA) or host metabolites to other compounds (e.g. secondary bile acids), as well as the inhibition of *C*. *difficile* by the production of primary microbial products (e.g. thuricin CD produced by *Bacillus thuringiensis*) [4, 7–10]. Indirect inhibition relates to microbiota-induced host immune reaction to control *C*. *difficile* colonization and proliferation. Microbe-associated molecular patterns (MAMPs) and danger-associated molecular patterns (DAMPs) induce either a homeostatic or an inflammatory response with involvement of adaptive (e.g. immunoglobulins) or innate (e.g. antimicrobial peptides) immune modulatory pathways [4, 11].

Among these mechanisms, the role of bile acids has been met with increasing interest. While primary bile acids like taurocholate can induce *C*. *difficile* germination, secondary bile acids like lithocholic and ursodeoxycholic acid (UDCA) have an inhibitory effect on germination of the bacteria [12, 13]. Recently, the *C*. *difficile* bile acid germinant receptor CspC, a germination-specific protease, has been identified as the link between bile acid-mediated germination and *C*. *difficile* infection in a hamster model [14].

The capability of bile acid transformation from primary to secondary bile acids is limited to a small subset of gut bacteria [15–17]. Within the *Clostridium* cluster XIVa, published evidence mainly involves *Clostridium scindens* and *Clostridium hylemonae* [18, 19]. Eight separate proteins are required to perform the bile acid biotransformation [20, 21]. One key enzyme of this secondary bile acid biosynthesis cascade is a stereo-specific NAD(H)-dependent 3-dehydro-4-bile acid oxidoreductase, encoded by the *baiCD* gene cluster, located within the *bai* operon [21, 22]. The level of the secondary bile acid deoxycholic acid is regulated by the concentration of bile acid 7α-dehydroxylating gut bacteria [23]. Within the *Clostridium* cluster XIVa, *C*. *scindens* and *C*. *hiranonis* have an at least 10 times higher 7α-dehydroxylating activity than other clostridial species [15, 24, 25]. Intriguingly, it has been reported that antibiotic therapy and subsequent disruption of the commensal microbiota lead to a decrease in the number of 7α-dehydroxylating bacteria, and to a shift of the fecal bile acid pool from secondary to primary bile acids [26–29]. In 2015, Buffie *et al*. identified several species (*Clostridium scindens*, *Pseudoflavonifractor capillosus*, *Barnesiella spp*. and *Blautia hansenii*) mediating CDI resistance in a mouse model, and corroborated these findings in a small number of hematologic patients [30]. In particular, *C*. *scindens* was negatively associated with CDI, which may be owing to its role in secondary bile acid biosynthesis.

Bile acid 7α-dehydroxylation via *baiCD* gene cluster encoded 3-dehydro-4-bile acid oxidoreductase is thus likely a decisive component of colonization resistance against *C*. *difficile*. Despite the high pathophysiological relevance demonstrated in preclinical studies, the role of this metabolic step in patients with *C*. *difficile* infection is largely unclear. The aim of the present study was to investigate the abundance of the *baiCD* gene cluster in patients with CDI or *C*. *difficile* colonization, and in controls as well as in patients undergoing fecal microbiota transplantation for recurrent CDI.

## Material and methods

### Sample collection

Fecal samples were collected from patients aged ≥ 18 years within 48 hours of admission to one of five German university-affiliated hospitals (Hannover, Tübingen, Munich, Cologne and Lübeck) and were stored directly at -80°C within a prospective, observational multicenter study (SPECTRUM), which aims at the identification of clinical and microbiota-associated risk factors for *C*. *difficile* infection (CDI) and colonization. The study was performed according to GCP guidelines as well as the ethical guidelines of the Declaration of Helsinki. All participants gave written informed consent. Detailed informations about the SPECTRUM study are given in the German Clinical Trials Register (www.drks.de, registered as DRKS00005335). Approval for the anonymous assessment of sample collection and patient data was granted by the ethics committees of all contributing study sites (internal approval number and approval data in parentheses): Hannover (No. 6444, 2013), Tübingen (No. 497/2013BO2, 2013), Munich (No. 5906/13, 2013), Cologne (No. 13–282, 2013) and Lübeck (No. 15–088, 2015).

In addition, out of the daily microbiological routine, 72 fecal samples of symptomatic patients tested positive for toxigenic *Clostridium difficile* (CDI) either by toxin B ELISA or by conventional culture were analyzed. These samples were negative for other tested pathogens (Salmonella spp., Shigella spp., Yersinia spp., Campylobacter spp.) to avoid bias in fulfilling the CDI case definition (diarrhea and positive toxin test, [31]). Approval for the anonymous assessment of sample collection and patient data was granted by the ethics committee of Hannover Medical School (No. 3378–2016, 2016).

### GDH test and *Clostridium difficile* toxin assay performance

For initial *Clostridium difficile* screening, 189 fecal samples from the SPECTRUM study were tested for GDH (glutamate dehydrogenase) antigen, which indicates the presence of *C*. *difficile* (toxin-positive and toxin-negative strains alike), by an EIA assay according to the manufacturer’s instructions (GDH EIA, C. DIFF CHEK^™^—60, Techlab, Blacksburg, USA). GDH EIA assays possess a sensitivity of 85%–95% and a specificity of 89%–99% [32, 33]. GDH test positive fecal samples were tested by PCR for toxin A (*tcdA*) and B (*tcdB*) to differentiate between patients colonized with non-toxigenic *C*. *difficile* (NTCD; GDH test positive, toxin negative) or patients asymptomatically colonized with toxigenic *C*. *difficile* (TCD; GDH test positive, toxin positive) [34–41]. PCR targeting of the toxin genes for toxin A (*tcdA*) and B (*tcdB*) was performed with a standardized in-house real-time PCR assay on a CFX96^™^ Real-Time PCR Detection System (Bio-Rad Laboratories, Hercules, CA, USA) for all stool samples of patients with positive GDH test [42, 43]. Toxin PCR positivity was defined as a positive result for *tcdA*, *tcdB*, or both. We note that the detection of the toxin genes cannot predict the expression of toxins in vitro or in vivo with certainty since the expression is controlled by multiple transcriptional regulators, including TcdR. Nevertheless, carriage of the toxin genes is a prerequisite and the most important known predictor of toxin production.

### DNA extraction from stool samples

DNA was extracted with the QIAcube DNA extraction system according to the manufacturer’s instructions (modified QIAamp DNA Mini kit protocol for tissue extraction (Qiagen, Hilden, Germany)) [44]. Briefly, approximately 100 mg stool was vortexed with 600 μl S.T.A.R. buffer (Roche, Mannheim, Germany) and 60 μl chloroform before being spun for 1 min. 200 μl of the supernatant was transferred into 2 ml microcentrifuge tubes, pre-filled with 0.1 mm (~0.5 g) and 0.3 mm (~0.35 g) beads and incubated with 500 μl buffer ASL for 5 min at 70°C. Homogenization was performed with Precellys24^™^ (Bertin Corp., Rockville, USA), followed by a centrifugation step for 2 minutes. 350 μl of supernatant was used for DNA extraction. The DNA was eluted with 2 × 50 μl aliquots of Buffer AE and stored at 4°C.

### *baiCD* gene cluster qPCR

*BaiCD* gene cluster qPCR was performed with a CFX 96^™^ Real-Time PCR Detection System (Bio-Rad Laboratories, Hercules, CA, USA) and Bio-Rad CFX Manager 3.1 Software. *BaiCD* gene cluster-specific primers were used to target *C*. *scindens* and *C*. *hiranonis* with modified primers based on the publication by Ou et al. [45]. These published *baiCD* gene cluster primer pairs were modified by aligning the *baiCD* genes DNA sequence from *C*. *scindens* VPI 12708 [46] and *C*. *hiranonis* TO-931 [16] using CLC genomics Workbench 8.5.1. (Qiagen, Hilden, Germany). In CLC genomics Workbench 8.5.1 alignment with annotated sequences of the *baiCD* genes (www.ncbi.nlm.nih.gov) of different strains (sequences of *C*. *scindens* VPI 12708, C. *scindens* VE202-05, *C*. *scindens* ATCC35704 (DSM 5676), *C*. *hiranonis* TO-931 (DSM 13275) and *C*. *hylemonae* TN271 (DSM 15053), were used to specify the published primers (S2 Fig). We sequenced *C*. *scindens* ATCC35704 (DSM 5676) and *C*. *hiranonis* TO-931 (DSM 13275). Our sequence of the *baiCD* genes in contig 6 was 100% identical with the published sequences (S2 Fig).

The specific primers including the forward primer *baiCD*-F (5′-CAGCCCRCAGATGTTCTTTG-3′) and the reverse primer *baiCD*-R (5′-GCATGGAATTCHACTGCRTC-3′) were identified to be the largest and most specific potential primers in the *baiCD* genes and were synthesized commercially (TIB Molbiol, Berlin, Germany). Performance and optimal annealing temperatures of the PCR primers were first tested with gradient PCR on the CFX 96^™^ Real-Time PCR Detection System (Bio-Rad Laboratories, Hercules, CA, USA). The highest primer efficiency was seen at 400 nM for the forward primer and 600 nM for the reversed primer. The cycling conditions were as follows: 95°C for 15 min followed by 45 cycles of 94°C for 15 s, primer-specific annealing temperature at 60°C for 30 s, and 72°C for 30 s. After amplification, a dissociation step was included to analyze the melting profile of the amplified products. Ten-fold dilution series of a specially designed gBlock Gene fragments (Integrated DNA Technologies, Coralville, IA) standard for the respective bacterial group or species were run along with the samples.

The gBlock Gene Fragments (Integrated DNA Technologies (IDT), Coralville, IA) containing the *baiCD* gene cluster qPCR target were purchased to estimate the detection limits of qPCR limits. A 10 fold serial dilution of the gBlock Gene Fragment (2 x 10^7^ to 2 x 10^0^ copies/μl per reaction) was run on every *baiCD* gene cluster qPCR assay to determine the slope. The prevalence of *baiCD*-positive samples was done by endpoint measurement. The fecal samples were counted as positive for *baiCD* when the reaction crossed the fluorescence threshold, a fluorescent signal significantly above the background fluorescence (usually 10 times the standard deviation of the baseline [47]). For quantification, the copy number had to be in the range of standard (ten-fold dilution series of a specially designed gBlock Gene fragments (IDT) standard for the respective bacterial group or species, which were run along with the samples). Four samples in the NC group, 1 sample in the NTCD and the TCD group and 2 samples in the CDI group were excluded due to low copy numbers from final analysis.

We controlled *baiCD*-negative fecal samples for PCR inhibitors, because inhibitors within the samples could interfere with the PCR by interacting directly with DNA, degrading or trapping the target DNA or blocking the activity of the polymerase. Common PCR inhibitors are the used material in the laboratory, food ingredients or the environmental background material [48–50]. For the detection of PCR inhibition, we used the housekeeping gene β-actin, which is a reliable internal reference gene. The gene-specific primers for β-actin for the internal control included Humu ACT_F (5´-CTTCAACACCCCAGCCATGT-3) and Humu ACT_2R (5´-TCTCCTTAATGTCACGCAGA-3). The mix included 10 μl of 2x Quanti-Tect SYBR Green Master Mix 0.3 μl of the forward and 0.3 μl of the reverse primer, 5 μl of target DNA and 4.4 μl of water. A known amount of positive control DNA is amplified with the sample, and inhibition is observed by a change in the quantitation of positive control DNA. Gastric cancer cell lines AGS were used as positive controls. The PCR conditions included: 95°C for 15 min followed by 45 cycles of denaturation at 94°C for 15 seconds, primer-specific annealing temperature at 60°C for 30 seconds, and an elongation step at 72°C for 30 seconds. The annealing temperature of 60°C was chosen because of required compatibility for *C*.*scindens* and *C*. *hiranonis* PCR assays. For our final analysis, we excluded samples which were *baiCD*-negative as well as negative for the internal reference housekeeping gene β-actin, in the assumption, that the inhibitors affecting the target DNA and the species-specific primers for the *baiCD* genes could not bind.

### DNA extraction from bacteria

The specificity of the *baiCD* gene cluster primers was confirmed against the non-target test bacteria; otherwise, results from the sequence searches against bacteria DNA databases were relied upon. The non-target test bacteria included *Streptococcus mitis* (in-house strain isolation out of the biliary tract), *Clostridium difficile* (ATCC 700057), *Escherichia coli* ATCC 25922, *Eubacterium aerofaciens* (in-house strain isolation out of douglas pouch), *Bacillus fragilis* ATCC 25285, *Clostridium perfringens* ATCC 13124, *Clostridium hylemonae* DSM 15053, *Clostridium sordellii* DSM 2141, *Lactobacillus rhamnosus* (in-house strain isolation out of feces), as well as *Blautia hansenii* DSM 20583. *Clostridium scindens* DSM 5676 and *Clostridium hiranonis* DSM 13275 were used as positive controls (S3 Fig). DNA extraction from bacteria was performed according to the manufacturer’s instructions (QIAamp DNA Mini kit protocol for bacteria, Qiagen, Hilden, Germany) [51]. Briefly, harvested bacteria were centrifuged for 10 min. The bacterial pellet was resuspended in 180 μl enzymatic lysis buffer (Qiagen, Hilden, Germany) and incubated for 30 min at 37°C. The next step included 25 μl of proteinase K (Qiagen, Hilden, Germany) and 200 μl AL buffer (Qiagen, Hilden, Germany) and mixed with the enzymatic lysis buffer. After this step, 200 μl ethanol (96%) was added to the sample and mixed thoroughly by vortexing. The precipitate mix was pipetted into the QIAamp DNA Mini spin column and centrifuged for 1 min. 500 μl Buffer AW1 (Qiagen, Hilden, Germany) was added and centrifuged for 1 min. 500 μl buffer AW2 (Qiagen, Hilden, Germany) was added and centrifuged for 3 min. The last step included the placement of the QIAamp DNA Mini spin column in a clean 1.5 ml or 2 ml microcentrifuge tube with 200 μl Buffer AE (Qiagen, Hilden, Germany) directly added onto the spin column membrane. The spin column was incubated at room temperature for 1 min and then centrifuged to elute. The purity and yield of the DNA were assessed spectrophotometrically (Nanodrop 1000; Thermo Fisher Scientific, Waltham, MA USA) by calculating the A260/A280 ratios to determine DNA concentrations.

### Quantification of 16S rDNA copy number density by rtPCR

As a proof of principle, to exclude a potential influence of stool consistency on the number of *baiCD* genes, we assessed the total bacterial concentration by 16S rDNA qPCR in a random number of stool samples (nine stool samples of Bristol Stool Scale (BSS) 1–4, 18 stool samples of BSS 5–6 and 19 stool samples of BSS 7). The BSS consists out of seven consistency categories of human feces, starting with low scores for hard stools and longer colon transit times whereas higher scores correspond to loose watery stools and fast transit times [52]. The first two types indicate constipation, 3 and 4 indicate formed/normal stool, 5 and 6 indicate unformed/liquid stool and 7 indicate diarrhea. We therefore phenotypically grouped the fecal samples into three groups: 1–4, 5–6 and 7 according to the part of water and solids in the stool. We used a previously published set including the forward primer Uni331F (5′-TCCTACGGGAGGCAGCAGT-3′), the reverse primer Uni797R (5′-GGACTACCAGGGTATCTATCCTGTT-3′) and the probe ((6-FAM)-5«-CGTATTACCGCGGCTGCTGGCAC-(3-BBQ)) for the 16S rDNA PCR [53]. Real-time PCR was performed in the LightCycler^®^480 Real-Time PCR System (Roche Diagnostic, Mannheim, Germany) and LightCycler 480 Software release 1.5.1.62. PCR reactions contained 18 μl of master mix and 2 μl of template DNA. The final reaction mixture contained: 8 μl of 2.5x Molzym Master Mix (Molzym GmbH & Co.KG, Bremen, Germany), 0.3 μM of the primers Uni331F and Uni797R, 0.15 μM of the probe, 0.8 μl of Molzym Polymerase, 100 μg/μl BSA (New England Biolabs) and 2 μl of template DNA. PCR conditions included an initial incubation step of 5 minutes at 95°C, followed by 45 cycles of denaturation at 95°C for 10 seconds, followed by annealing and elongation at 60°C for 60 seconds. The amplification protocol ended with a cooling period of 40°C for 30 seconds.

### Reproducibility

For the preparation of the 16S rDNA gene standard *Escherichia coli* DNA was used for determining bacterial number by real-time PCR. The set included the forward primer 285F 5´-GAG AGT TTG ATC CTG GCT CAG–3` and the reverse primer 261R 5´-GAG GTG ATC CAG CCG CA-3´. A 1533 bp sized amplicon was generated via PCR. The PCR reactions contained 45 μl of master mix and 5 μl of template DNA. The final reaction mixture contained: 5 μl of 10x Buffer (18 mM MgCl_2_), 1 μl of dNTPs (10 mM each), 2 μl of 285F (10 pmol/μl), 2 μl of 261R (10 pmol/μl), 0,5μl of Faststart HiFi Polymerase Roche (5U/μl), 34,5 μl of water and 5 μl of DNA. The copy number was calculated using Avogadro’s number. Standards were serially diluted, extracted as described above, and subjected to the 16S PCR assay by two different technicians as well as the patient samples on three separate days. Non-template control (NTC) were included in all tests (S1 Fig).

### Donor selection, stool preparation and fecal microbiota transfer (FMT)

Out of routinely performed FMTs for recurrent *C*. *difficile* infection, we analyzed fecal samples of two patients before and after FMT. Donor selection was performed in accordance with the criteria of Paramsothy et al. [54] and approved by the local ethics committee (request of expanded access for each patient (30.03.2015)). Fresh donor stool was processed not later than 2 h after collection, and the transfer to the recipient was performed within 6 h after stool donation. In accordance with the systematic review of Cammarota et al. [55] and the published statement of the European consensus conference on fecal microbiota transfer [56], we collected approximately 100 g of donor fecal material and transferred it into a standard commercial blender. Subsequently, 200 ml of sterile normal saline (0.9% sodium chloride) were added, and the blender content was homogenized. The resulting slurry was filtered through different sized sieves and transferred in five 50 ccm syringes. The supernatant was administrated directly via colonoscopy in the ascending part of the colon in our endoscopy unit. The patients were prepared with local standard medication for colonoscopy (Oralav^®^; Moviprep^®^) and in the morning of FMT, the patients received loperamide to decrease large bowel movements. Any previously administered antibiotic treatment was stopped 48 h before FMT.

### Statistical analysis

Differences in *baiCD* gene cluster abundance between the four study groups (NC, NTCD, TCD and CDI) were assessed using the chi-square test for categorical variables (binary variable or dichotomous variable, “yes” or “no” indicating the presence or absence of the *baiCD* genes). Post-hoc analysis was performed by pairwise comparisons using Fisher’s exact test. In addition, categorical variables from the table of baseline patients’ characteristics were used for statistical analysis, i. e. gender (male or female), the presence or absence of diarrhea, CDI, antibiotics or proton-pump inhibitors. For analyzing the presence or absence of the *baiCD* genes in fecal samples, we used a double approach. If the results were inconsistent, we would re-run the PCR protocol with this sample. The reproducibility of the 16S rDNA PCR was proven in 4 different patient samples by two different technicians on three separate days (S1 Fig). One-way ANOVA was performed on the gene copy numbers of the different groups. Unpaired t-test was performed for the relative abundance of *baiCD* genes in fecal samples. Data were displayed in box-and-whiskers plots showing the median, interquartile range, and range from minimum to maximum.

All p values were two-sided and the level of significance was set at p<0.05. In case of pair-wise comparison within the four groups, the p-value was not adjusted for multiple testing as we assumed our analysis being hypothesis generating. All data were analyzed using GraphPad Prism 6.0 (GraphPad Software Inc., La Jolla, CA, USA).

## Results

### Patient characteristics

Overall, four sample groups were available for further analysis (NC (n = 148), NTCD (n = 14), TCD (n = 27) and CDI (n = 72)). GDH testing revealed that 138 samples were negative for *C*. *difficile* (non-colonized; NC), and 33 were positive; *C*. *difficile* toxin PCR was positive in 21 of the latter samples (toxigenic *C*. *difficile*; TCD) and negative in 12 (non-toxigenic *C*. *difficile*; NTCD). 18 samples were excluded due to PCR inhibition or missing data. To obtain data from patients with symptomatic CDI, we included 64 patients who were diagnosed with CDI in clinical routine testing (diarrhea and *Clostridium difficile* toxin ELISA and/or standard toxigenic culture technique positive). 8 samples had to be excluded due to PCR inhibition or missing data.

There were significantly more females in the CDI group compared to the TCD group (Table 1, p<0.01). The age between the groups was similar. As expected, CDI patients suffered from recent diarrhea more often than NC, NTCD or TCD patients (p<0.001). There was no difference in the previous history of CDI between the groups. Previous exposure to antibiotics was least common in the NC group as compared to the NTCD group (p<0.05), the TCD group (p<0.05) or the CDI group (p<0.01). No difference in the use of acid suppressive drugs was observed between the groups.

**Table 1:** Baseline characteristics.

Patient characteristics	NC (n = 138)	NTCD (n = 12)	TCD (n = 21)	CDI (n = 64)	TOTAL (n = 235)
**Female (n, %)**	72 (52.2)	5 (41.7)	6 (28.6)	39 (60.9)*	122 (51.9)
**Age (years, mean±SD)**	61.2±14.96	57.67±15.88	63.57±12.11	62.62±19.42	61.6±15.98
**Diarrhea (≤3 months; n, %)**	34 (24.6)	4 (33.3)	6 (28.6)	64 (100.0)#	108 (46.0)
**Past CDI episode (≤3 months; n, %)**	3 (2.2)	0 (0.0)	0 (0.0)	2 (3.1)	5 (2.1)
**Antibiotics (≤3 months; n, %)**	38 (27.5)	7 (58.3)**	11 (52.4)¶	25 (39.1)§	81 (34.5)
aminoglycoside	0 (0)	0 (0)	1 (9.1)	1 (4)	2 (2.5)
penicilline±beta-lactamase inhibitors	14 (36.8)	2 (28.6)	5 (45.5)	13 (52)	34 (42)
carbapenem	3 (7.9)	1 (14.3)	5 (45.5)	7 (28)	16 (19.8)
cephalosporin	5 (13.2)	1 (14.3)	2 (18.2)	9 (36)	17 (21)
quinolone	13 (34.2)	3 (42.9)	4 (36.4)	8 (32)	28 (34.6)
colistin	0 (0)	0 (0)	0 (0)	1 (4)	1 (1.2)
glycopeptide	1 (2.6)	0 (0)	3 (27.3)	7 (28)	11 (13.6)
glycoside	2 (5.3)	0 (0)	0 (0)	3 (12)	5 (6.2)
glycylcycline	1 (2.6)	0 (0)	0 (0)	0 (0)	1 (1.2)
lincosamide	0 (0)	0 (0)	0 (0)	2 (8)	2 (2.5)
oxazolidone	0 (0)	0 (0)	2 (18.2)	1 (4)	3 (3.7)
macrolide	0 (0)	2 (28.6)	2 (18.2)	3 (12)	7 (8.6)
nitroimidazole	9 (23.7)	1 (14.3)	2 (18.2)	4 (16)	16 (19.8)
sulfonamide	8 (21.1)	1 (14.3)	1 (9.1)	1 (4)	11 (13.6)
unknown	0 (0)	1 (14.3)	0 (0)	0 (0)	1 (1.2)
**PPI (≤3 months; n, %)**	90 (65.2)	8 (66.7)	12 (57.1)	26 (40.6)	136 (57.9)

PPI: Proton pump inhibitor; SD: Standard deviation;

*: p<0.01 (TCD vs. CDI);

^#^: p<0.001 (NC/TCD/NTCD vs. CDI);

**: p<0.05 (NC vs. NTCD);

^¶^: p<0.05 (NC vs. TCD);

^§^: p<0.01 (NC/NTCD vs. CDI)

### Prevalence and relative abundance of the *baiCD* gene cluster

**Fig 1:**
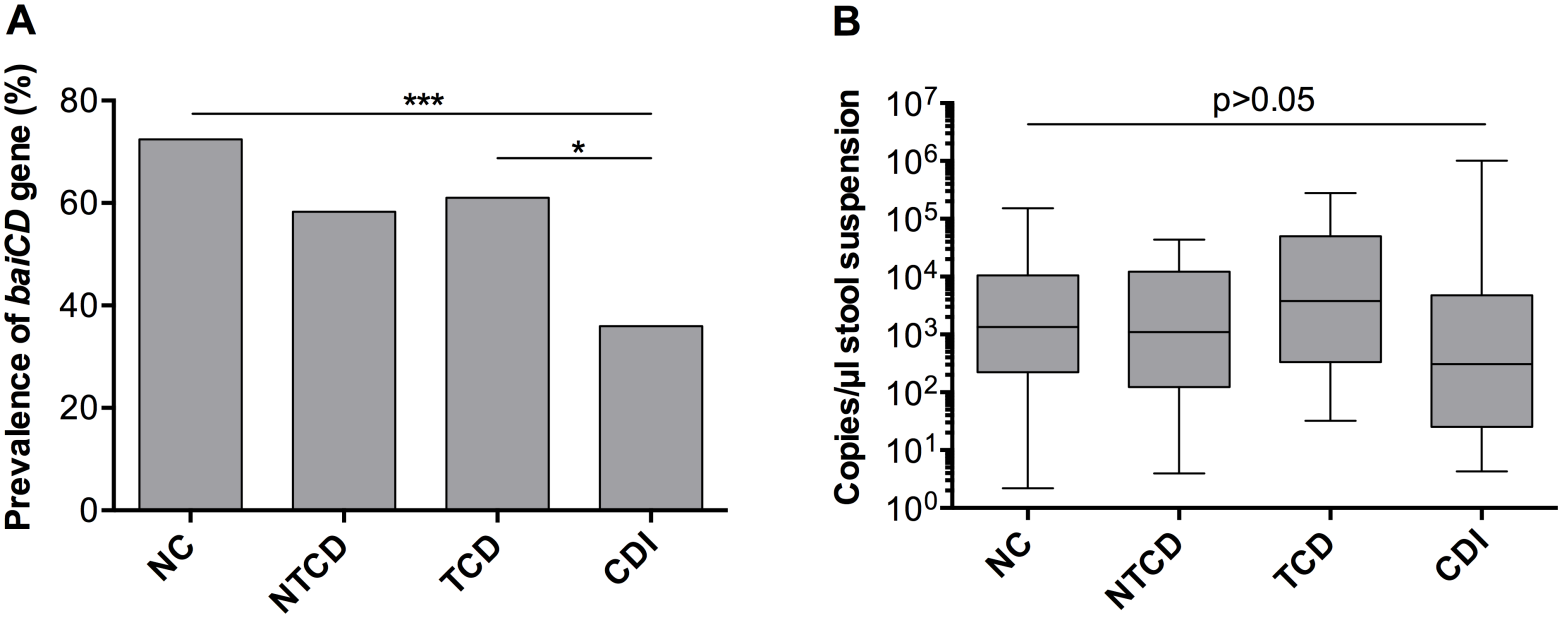
*BaiCD* gene cluster prevalence/abundance and *C*. *difficile* state. The prevalence/abundance of the *baiCD* gene cluster was analyzed in fecal samples of patients with *C*. *difficile* infection (CDI, n = 64), colonization with non-toxigenic *C*. *difficile* (NTCD, n = 12), toxigenic *C*. *difficile* (TCD, n = 21), and of *C*. *difficile*-negative (NC, n = 138) patients by species-specific qPCR. **Fig 1A**: End-point qPCR revealed a significantly higher prevalence of *baiCD* genes in NC and TCD than in CDI samples (*** p<0.0001, * p<0.05, chi-square test and Fisher´s exact test). **Fig 1B**: *BaiCD* gene cluster copy numbers were not statistically different in *baiCD*-positive NC (n = 96), NTCD (n = 6), TCD (n = 12) and CDI (n = 21) samples (box-and-whiskers plot, median-IQR-min/max, one-way ANOVA, p = n.s. (non-significant)).

Endpoint PCR measurements for detecting the *baiCD* genes showed *baiCD* positivity rates of 72.5% (100/138) in NC, 58.3% (7/12) in NTCD, 61.9% (13/21) in TCD and 35.9% (23/64) in CDI samples (Fig 1A). Comparing the prevalence of *baiCD*-positive fecal samples of asymptomatic (NC, NTCD, TCD) and symptomatic (CDI) patients, the prevalence was significantly higher in the NC group (p < 0.0001) and in the TCD group (p = 0.0447) compared to the CDI group (Fig 1A). In comparison, the prevalence of *baiCD*-positive fecal samples did not significantly differ between colonized (NTCD and TCD) and non-colonized (NC) patients. Furthermore, no differences were seen between colonization with a toxigenic or non-toxigenic strain.

To obtain an estimate of the abundance of *baiCD*-positive bacteria in the samples tested positive for the *baiCD* genes, copy numbers were compared (Fig 1B). Quantification of the *baiCD*-positive samples showed no significant differences between the three asymptomatic groups and CDI (p = 0.2827, Fig 1B). The median copy number was 1.3 x 10^3^ copies/μl stool suspension for NC (min. 2.19 x 10^0^ to max. 1.5 x 10^5^), 1.1 x 10^3^ copies/μl stool suspension for NTCD (min. 3.9 x 10^0^ to max. 4.4 x 10^4^), 3.8 x 10^3^ copies/μl stool suspension for TCD (min. 3.2 x 10^1^ to max. 2.8 x 10^5^) and 3.1 x 10^2^/μl stool suspension (min. 4.3 x 10^0^ to max. 1 x 10^6^) for CDI (Fig 1B).

**Fig 2:**
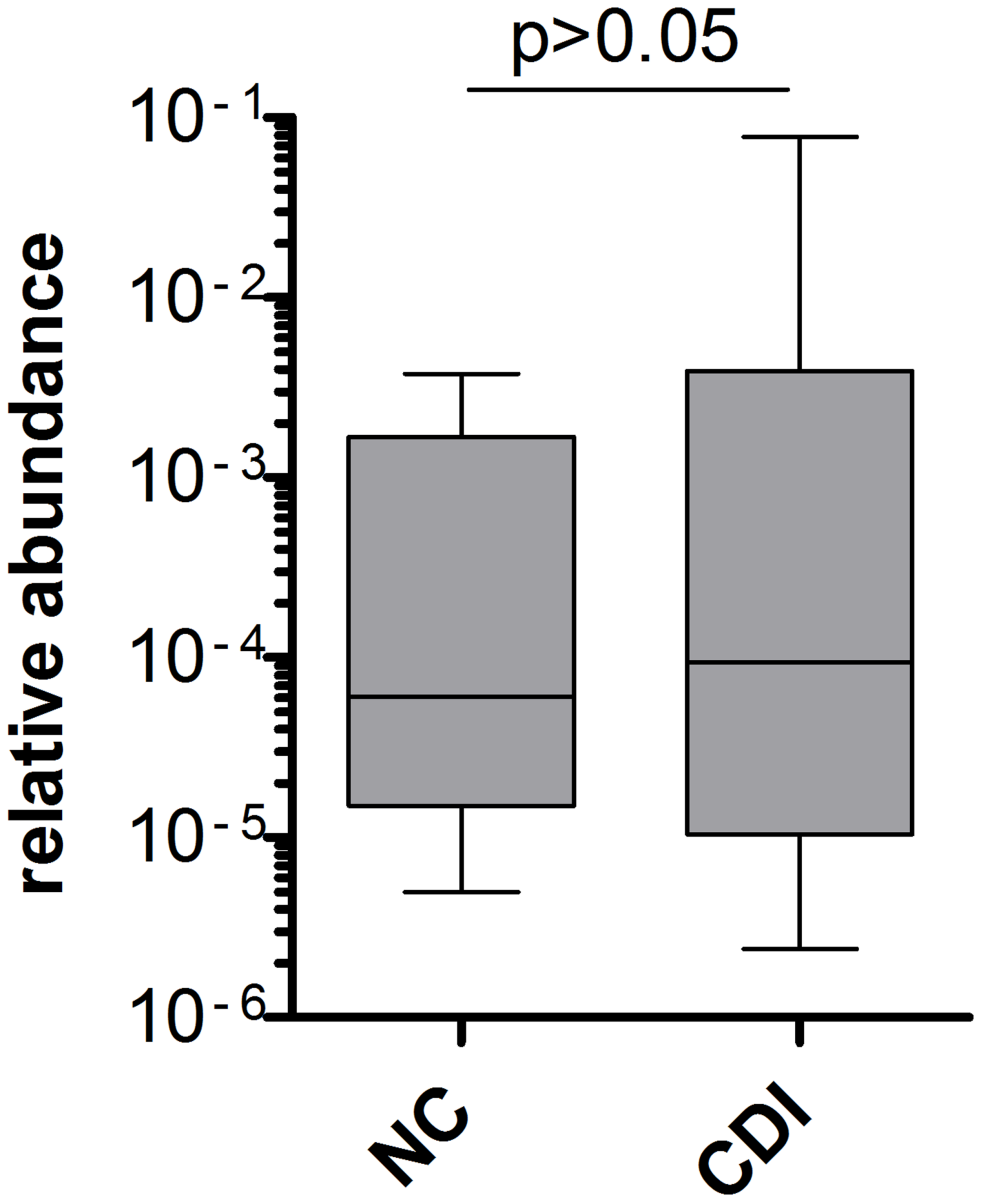
*BaiCD* gene cluster abundance in relation to the total bacterial load. The ratio *baiCD*/16S rDNA was determined for a subset of *baiCD*-positive fecal samples. No significant difference in the relative *baiCD* gene cluster abundance between NC (*C*. *difficile* negative) and CDI (*C*. *difficile* infection) samples could be detected (n = 9 in each group; box-and-whiskers plot, median-IQR-min/max, one-way ANOVA, p = n.s.).

Next, we sought to evaluate the abundance of *baiCD* genes in relation to the total bacterial load. For a subgroup of *C*. *difficile*-negative and CDI patients who were *baiCD*-positive, the *baiCD* gene cluster copy number was compared to the total 16S rDNA copy number as a measure for the total bacterial load and the relative abundance of *baiCD* genes was calculated (Fig 2). The ratio *baiCD*/16S rDNA was determined for each individual sample and the median was computed for each subject group. There was no significant difference between the relative abundances of both groups (p = 0.3244). The median of the NC group was 6.03x10^-5^ (4.97x10^-6^–3.76x10^-3^) and for the CDI group 9.44x10^-5^ (2.4x10^-6^–7.81x10^-2^). This indicates that when *baiCD* gene cluster is detectable, there is no difference in relative *baiCD* gene cluster abundance between *C*. *difficile* negative and CDI fecal samples.

### 16S rDNA quantification in different stool consistencies

**Fig 3:**
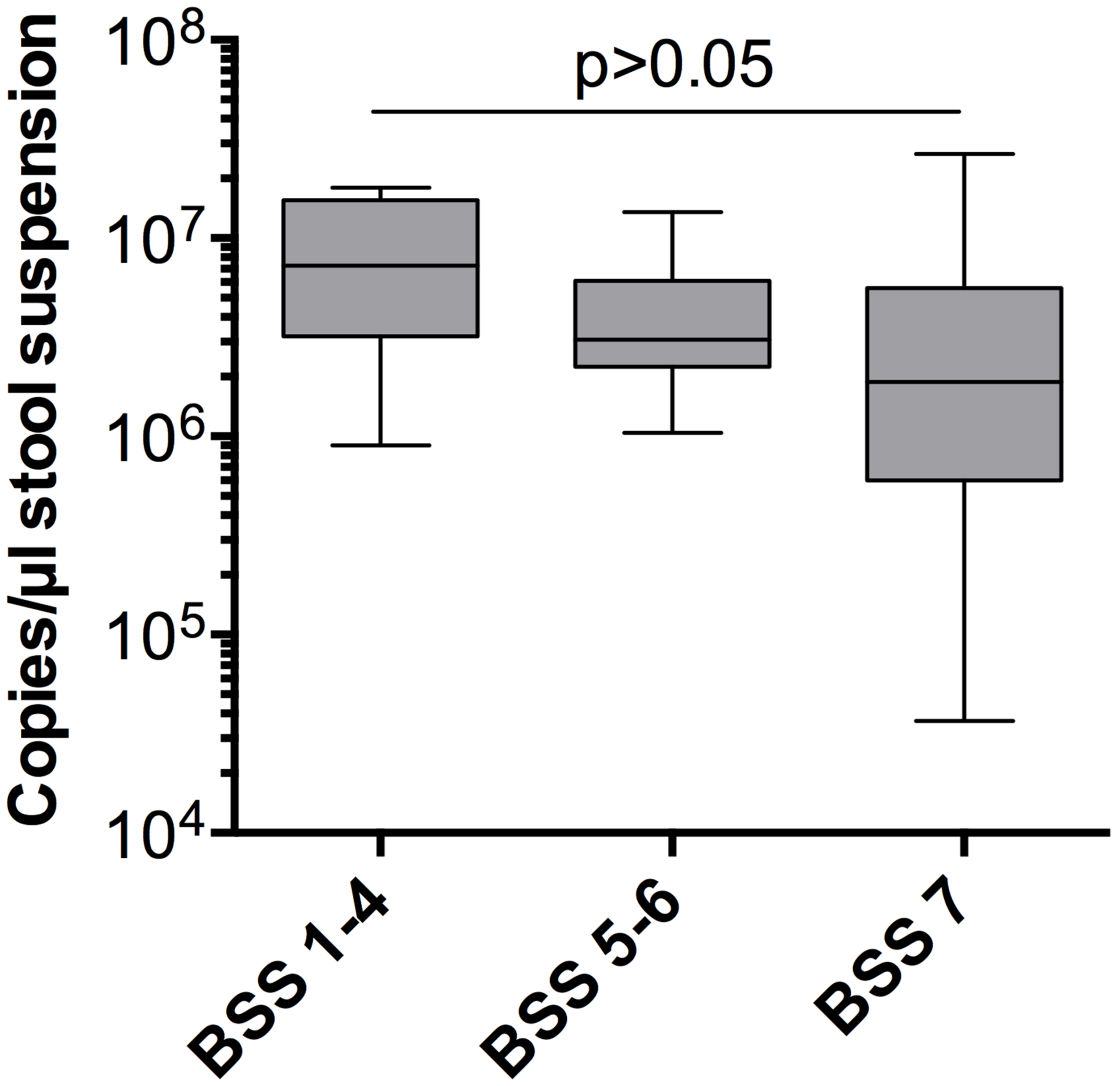
Total bacterial concentration and stool consistency. 16S rDNA qPCR quantification as a measure of the total bacterial concentration was carried out in samples of three different stool consistencies, namely BSS (Bristol Stool Scale) 1–4 (constipated/normal), 5–6 (unformed/liquid), and 7 (diarrhea). 16S rDNA abundance was not statistically different between the groups (box-and-whiskers plot, median-IQR, one-way ANOVA: p = 0.1194).

When comparing asymptomatic and CDI patients, different stool consistencies have to be anticipated. To examine whether stool consistency affects the total bacterial concentration, we performed 16S rDNA qPCR in stool samples of different consistencies [Bristol stool scale (BSS) 1–4, BSS 5–6 and BSS 7]. For proof of principle, we tested nine stool samples of BSS 1–4, 18 stool samples of BSS 5–6 and 19 stool samples of BSS 7 (Fig 3).

The median of the nine stool samples with a BSS 1–4 was 7.25 x 10^6^ copies/μl stool suspension (min. 9 x 10^5^ to max. 1.8 x 10^7^), the median of the 18 stool samples with a BSS 5–6 was 3.09 x 10^6^ copies/μl (min. 1.04 x 10^6^ to max. 1.35 x 10^7^), and the median of the 19 stool samples of BSS 7 was 1.9 x 10^6^ copies/μl (min. 3.7 x 10^4^ to max. 2.7 x 10^7^). The widest range of copy numbers was seen in the patient samples with a BSS of 7. There was no significant difference between the copy numbers in different stool consistencies (p = 0.1194). This indicates that stool consistency does not measurably influence the relative abundance of bacterial species in this context of complex microbial communities.

### FMT cases, outcome and the occurrence of *baiCD* genes

Given the high risk of recurrence and the importance of microbiome perturbances for the pathogenesis of CDI, fecal microbiota transplantation has been associated with high success rates in several clinical studies on patients suffering from rCDI, and incorporated in international CDI guidelines [31, 57].

Patient 1 was a 35-year-old Caucasian female who presented with multiple episodes of recurrent CDI due to multiple antibiotic usage for several reasons over a 6-month period (confirmed by positive *C*. *difficile* ELISA testing). Sequential treatment with metronidazole, vancomycin, and fidaxomicin had failed. The patient was treated with 250 ml fecal suspension of the patient’s spouse via colonoscopy. She could be discharged on the same day and was symptom-free after three days. During a followed-up period (3 months and one year later), the patient was still *C*. *difficile* toxin negative.

Patient 2 was a 72-year-old Caucasian female who presented with multiple episodes of recurrent CDI after treatment with clindamycin for erysipelas. At initial diagnosis, the patient suffered from pseudomembranous colitis with bloody-slimy diarrhea 10 times daily and abdominal cramps. All approved antibiotics for treatment of CDI failed. We performed FMT with 250 ml fecal suspension of the patient’s son-in-law via colonoscopy. After FMT, the patient immediately was symptom-free. During a followed-up period (11 days and 42 days later), the patient was still *C*. *difficile* toxin negative and free of symptoms.

The FMT procedure was well tolerated in both patients and no adverse or severe adverse events were noticed. Since patient two came from a more distant location and was completely symptom-free after the treatment, the pre-scheduled follow-up visit was missed.

**Fig 4:**
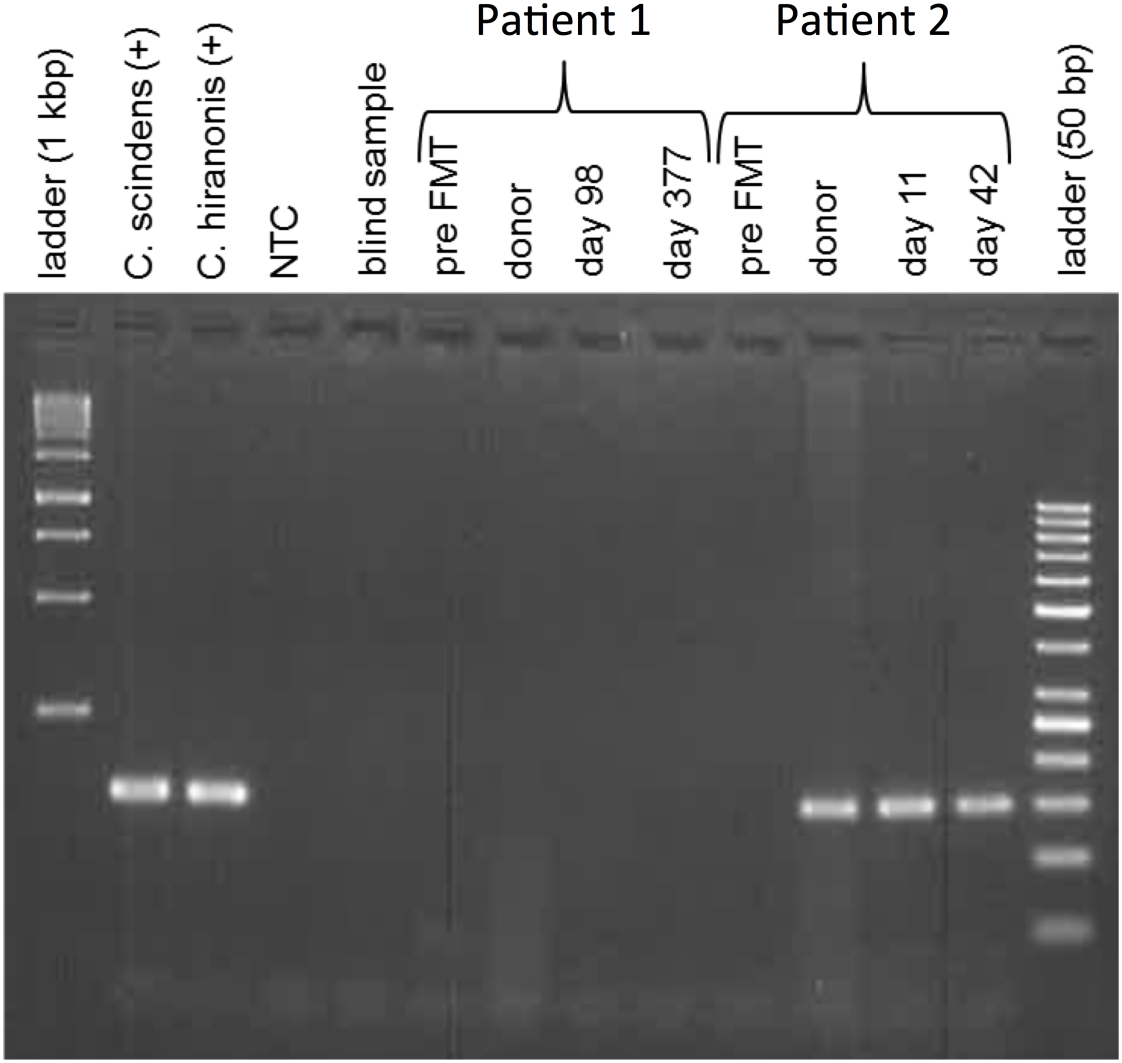
Presence of the *baiCD* gene cluster in 2 patients before and after fecal microbiota transplantation (FMT). Agarose gel loaded with products of an end-point PCR detecting the *baiCD* gene cluster (92 bp) of *C*. *scindens* and *C*. *hiranonis* (positive control, lane 2+3; negative controls: lane 4+5). In patient 1 (lanes 6–9), *baiCD* gene cluster was neither detected before, nor after FMT or in the donor. Patient 2 (lanes 10–13) was *baiCD*-negative before the procedure, but persistently positive after receiving FMT from a *baiCD*-positive donor. (Lane 1+14: 1 kbp and 50 bp DNA ladder; NTC = non-template control)).

We performed qPCR for the *baiCD* genes in fecal samples of the patients before FMT and during the follow-up period as well as of the donor at baseline and performed agarose gel electrophoresis of the qPCR products (Fig 4). Patient 1 showed no *baiCD* positivity at baseline and during follow-up. The patient’s donor was *baiCD*-negative as well. Patient 2 was *baiCD*-negative at baseline and became *baiCD*-positive during follow-up. The donor was *baiCD*-positive.

## Discussion

Intestinal dysbiosis can disrupt mechanisms of CDI resistance, which is thought to include a decrease of bile acid 7α-dehydroxylating microbial species, leading to reduced secondary bile acid biosynthesis [58–60]. The genes required for this metabolic step have been located in a single bile acid inducible (*bai*) operon in *C*. *scindens* as well as in *C*. *hiranonis*. This is the first study investigating the relationship of the *baiCD* gene cluster and *C*. *difficile* colonization/infection in human fecal samples.

Our experiments show that we could not only identify the *baiCD*-positive species *C*. *scindens* and *C*. *hiranonis* in a species-specific PCR, but also quantify the copy numbers in just one run. The specificity of our *baiCD* gene cluster PCR primer pair was verified by detecting a single PCR product using DNA from bile acid 7α-dehydroxylating bacterial strains (S3 Fig) and extensive sequence comparisons with sequence databases.

It is known that in the setting of a disrupted microbiota during *C*. *difficile* infection, the number of bile acid 7α-dehydroxylating microbial species is decreased with consecutively reduced secondary bile acid concentrations and increased primary bile acids as a potent germinant for *C*. *difficile* [12, 58–61]. However, data on the prevalence of *baiCD*-positive species in fecal samples is sparse. Kitahara et al. examined 34 samples of healthy individuals using direct PCR. The authors found *C*. *scindens* in 27, and *C*. *hiranonis* in 18 samples [62]. Recently, an association between colonization resistance to *C*. *difficile* and the presence of *C*. *scindens* (among other species) was reported on the basis of a murine infection model and a small number of hematologic patients (n = 12) [30]. However, evidence on the prevalence of *baiCD*-positive species in the context of *C*. *difficile* colonization or infection, and during FMT in patients—both of which would potentially provide clues regarding their relevance for *C*. *difficile*-associated colonization resistance—is lacking. In our study, the prevalence of *baiCD* positivity was indeed higher in non-CDI samples as compared to CDI samples. It has to be kept in mind, however, that we analyzed patients admitted to the hospital, and that the prevalence of the *baiCD* gene cluster may be influenced by pre-treatments (e.g. antibiotics, immunosuppression), pre-hospitalization as well as by different diseases. There were no significant differences in the prevalence of the *baiCD* genes between NC, NTCD and TCD samples, even though the prevalence was numerically higher in NC samples than the colonized ones. Contrary to our expectations, significant differences in the prevalence could be seen between TCD and CDI. However, the number of GDH positive and toxin PCR positive non-CDI-samples was too small to draw firm conclusions. The idea that *baiCD*-carrying bacteria play a role not only in *C*. *difficile* infection but also in colonization is intriguing and has to be tested in a larger sample.

Recently, Studer et al. showed that in gnotobiotic mice harboring a simplified murine 12-species "oligo-mouse microbiota", which represents the major murine intestinal bacterial phyla, but is deficient for 7α-dehydroxylation, the addition of *C*. *scindens* significantly decreased *C*. *difficile* colonization and pathogenesis [63]. 24h and 72h after infection with *C*. *difficile*, *C*. *scindens* and *C*. *difficile* densities as well as the cecal luminal bile acid composition were measured. 24 h post infection the *C*. *scindens*-associated mice showed partial resistance against *C*. *difficile* colonization associated with the secondary bile acid deoxycholic acid (DCA) in the cecal contents, whereas 72 h post infection, a complete loss of protection was observed, associated with a near-complete absence of microbial bile acid transformation products. At this time point, median *C*. *scindens* densities were decreased, but not zero.

These data seem to indicate that not only the presence but also the abundance of *C*. *scindens* could play a role in infection resistance [30]. We therefore analyzed the *baiCD* gene cluster in comparison to the total 16S rDNA copy number in the *baiCD*-positive samples of each group. Overall, the relative *baiCD* abundance was not significantly different between *C*. *difficile* negative and CDI fecal samples (Fig 2). Our results thus support the notion that the relevant factor for *C*. *difficile* resistance is the mere presence of the *baiCD* gene cluster, while the relative gene copy number seems less important. In the study by Studer and colleagues, the addition of *C*. *scindens* had only minor effects on total bacterial composition [63]. This is in line with the estimate that only a small number of fecal bacteria (~0.0001%) have the ability to convert bile acids [17, 19, 64, 65].

Thus, our measurements reflect only a small number of species and focus on one special enzyme in a complex pathway of bile acid conversion. The total number of bacteria involved in secondary bile acid transformation can be expected to be higher. Furthermore, the number of 7α-dehydroxylating bacteria could be influenced—besides by antibiotic-induced microbiota disruption—by concomitant diseases. Berr et al. could show that cholesterol gallstone patients had approximately 1,000-fold higher fecal levels of 7α-dehydroxylating bacteria with high levels of secondary bile acids compared to patients with lower deoxycholic acid levels [23]. Besides cholesterol gallstones, it was demonstrated that patients with colon cancer had higher levels of fecal bile acids and concentrations of 7α-dehydroxylating clostridia in their stool [66, 67]. Within bacteria with 7α-dehydroxylating activity, Doerner et al. could show that these bacteria could be divided into high and low activity groups with an activity range greatly (>100-fold) among different strains [24]. For example, *C*. *sordellii* belonged to the group of “low activity” strains, whereas species such as *C*. *scindens* had 100-fold greater bile acid 7α-dehydroxylating activity *in vitro*.

As to a potential bias introduced by the different stool consistencies present in our samples, reliable data on the total bacterial concentrations in the presence and absence of diarrhea using modern molecular methods has not been published. A study in IBS-D patients revealed a somewhat reduced abundance of aerobic vs. anaerobic bacteria [68]. In a small series of ETEC-infected patients, no consistent changes in the total number of 16S rRNA sequences during the diarrheal episode were observed [69]. Rather than on the total bacterial load, several studies focused on the microbial diversity in diarrheal samples: Vandeputte et al. observed that stool consistency is associated with fecal microbial richness with the lowest in diarrheal samples [70]. Our data demonstrated that stool consistency in our cohort does not have any influence on the total 16S rDNA copy number. However, different numbers of copies of the 16S rRNA gene can occur in each bacterium, and this number can also vary between different phyla. There are species with only one copy of this gene, whereas other species harbor up to 15 copies of the 16S rRNA gene [71]. Indeed, we could not make a statement about bacterial richness but supporting our findings, Tigchelaar et al. did not confirm the results of Vandeputte et al. association of BSS with microbial richness in a larger cohort [72].

Fecal microbiota transplantation is known to support the correction of the bile acid imbalance occurring in rCDI patients [73]. We therefore hypothesized that the recovery from rCDI is associated with an acquisition of *baiCD* genes, and analyzed the prevalence of *baiCD* genes in two patients who underwent FMT for rCDI. Both patients were *baiCD*-negative at baseline (before FMT), but one patient became *baiCD*-positive at the least on day 11 after FMT. Interestingly, this patient’s donor was *baiCD*-positive, and may therefore have transferred *baiCD*-positive species (in our case *C*. *scindens* and *C*. *hiranonis*) to the transplanted individual. In 2015, Weingarden and colleagues examined the short- and long-term bacterial composition following FMT for rCDI [74]. She found that while the microbiota of the recipients’ samples was highly correlated to the microbiota of the donors’ samples on day 1, there was high fluctuation within and across patients after the initial time point. Khoruts et al. showed that by 14 days post transplantation, the fecal bacterial composition of the recipient was highly similar to that of the donor and was dominated by *Bacteroides* spp. strains and an uncharacterized butyrate producing bacterium [75]. While these and other published data indicate that the probability of *baiCD* gene cluster transfer in our patient was high, the future development of the individual microbiota is not well predictable, as it involves the pattern of the donor microbiome, but also the recovering recipient’s microbiome. A long time follow up will be needed to clarify whether the transferred *baiCD*-positive species will stay or disappear over time. Obviously, we do not have fecal samples of this patient representing the baseline situation, i. e. the microbiota composition before the occurrence of CDI (and FMT). It is theoretically conceivable that the patient was *baiCD*-positive before developing rCDI, that the respective species were suppressed due to the infection, and that afterward her microbiota recovered, leading to the detection of *baiCD*. Furthermore, it is certainly possible that FMT led to the (re-) appearance of other or additional species mediating CDI resistance [30, 76, 77]. Since we analyzed only fecal samples of two patients, the results must be interpreted carefully. For more statistical power, a larger cohort of fecal samples of patients undergoing FMT is required.

While there is a solid body of evidence pointing to a key role of bile acid metabolism in this context, Lawley et al [78] restored the microbiota in mice after antibiotic treatment and *C*. *difficile* infection with a mixture of six phylogenetically diverse intestinal bacteria, and demonstrated that 7α-dehydroxylating species are not a *conditio sine qua non* to resolve relapsing *Clostridium difficile* infection. In support of this, the work of Studer et al. showed that a complex consortium of bacteria and not only some species are responsible for CDI resistance [63, 78]. Together with published evidence, our results thus indicate that *baiCD*-positive species with the ability to mediate the conversion of primary to secondary bile acids may be one, but not the only factor in the incompletely understood system of CDI resistance. To add even more to the complexity, a recently published work showed that sterile fecal filtrate is able to induce CDI resistance or resolve CDI symptoms [79].

One limitation of our study is the heterogeneous sample size of the different groups, which may lead to statistical bias. On the other hand, this represents the distribution of non-colonized, colonized and toxin PCR positive individuals in an unselected cohort of patients admitted to the hospital. While the cases presented here offer the perspective that *baiCD* positivity could be transferred via FMT, this will have to be confirmed in a larger sample.

### Conclusion

In conclusion, our study shows that fecal samples of CDI patients are less frequently *baiCD*-positive than samples from *C*. *difficile*-negative individuals. This supports the hypothesis that *baiCD*-positive species are involved in the complex framework of *C*. *difficile* colonization resistance.

## Supporting information

S1 Fig Reproducibility of 16S rDNA copy number density by rtPCR. **S1A Fig**: A serial dilution starting with 10^8^ copies to 10^2^ copies of the standard (complete 16S gene *Escherichia coli* ATCC 25922) was used to determine the reproducibility of the 16S PCR assay as a double determination on three different days. Non-template controls (NTC) were included in all tests. The repeated measurements demonstrated a high reproducibility, independent of the technicians and sample types. **S1B Fig** shows the Ct values of four different fecal samples, measured as a double determination on three different dates.
(TIFF)

S2 Fig Alignment with annotated sequences of the *baiCD* genes. Pairwise comparison of different *baiCD* gene cluster sequences. Red colors showed highly (> 96%) matched sequences in comparison to the less well matched blue fields of sequences. The strains of *C*. *scindens* VPI 12708 and *C*. *scindens* VE202-05 were different, but the *baiCD* gene cluster sequence is 100% consistent.
(TIF)

S3 Fig Establishment of a species-specific primer PCR against non-target test bacteria. **S3A Fig**: Cross-reaction experiments with 10 ng DNA of different bacterial strains (red) demonstrated primer specificity for the *baiCD* genes of *C*. *scindens* (blue) and *C*. *hiranonis* (blue) with Ct values of 16.64 (*C*. *scindens*) and 18.01 (*C*. *hiranonis*), respectively. Yellow: Non-template control (NTC), orange: Blind sample, dark green: Automatically calculated threshold. **S3B Fig**: The corresponding melting curves for this experiment showed slightly different melting peaks for *C*. *hiranonis* and *C*. *scindens*.
(TIFF)
