# Supplementary figures and images for: BaiCD gene cluster abundance is negatively correlated with Clostridium difficile infection
Source: PLoS One. 2018 May 8;13(5):e0196977. doi: 10.1371/journal.pone.0196977 (PMC5940204; doi:10.1371/journal.pone.0196977)

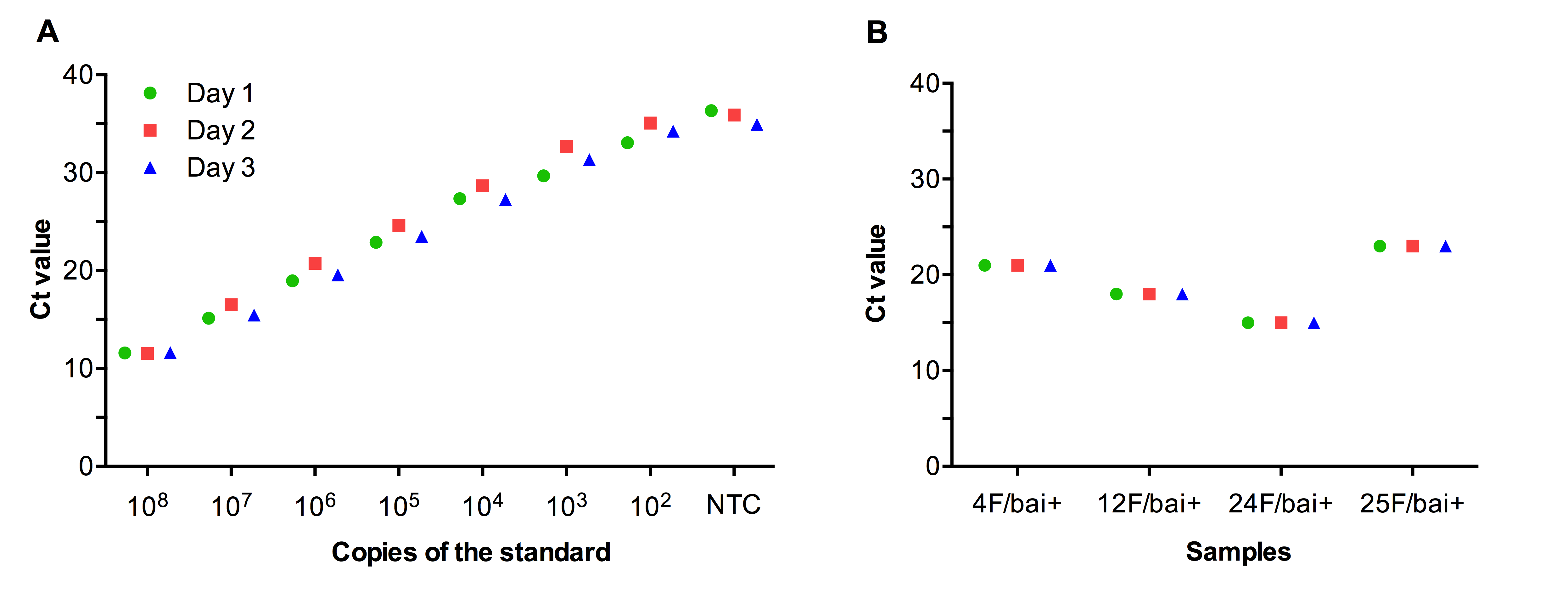

Supplement: S1 Fig — S1A Fig: A serial dilution starting with 108 copies to 102 copies of the standard (complete 16S gene Escherichia coli ATCC 25922) was used to determine the reproducibility of the 16S PCR assay as a double determination on three different days. Non-template controls (NTC) were included in all tests. The repeated measurements demonstrated a high reproducibility, independent of the technicians and sample types. S1B Fig shows the Ct values of four different fecal samples, measured as a double determination on three different dates. (TIFF) [file pone.0196977.s001.tiff]

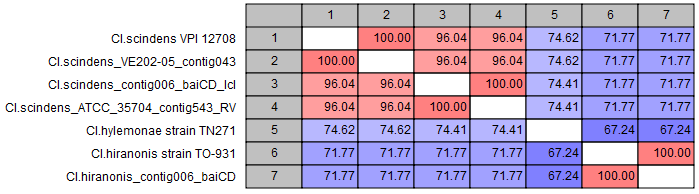

Supplement: S2 Fig — Pairwise comparison of different baiCD gene cluster sequences. Red colors showed highly (> 96%) matched sequences in comparison to the less well matched blue fields of sequences. The strains of C. scindens VPI 12708 and C. scindens VE202-05 were different, but the baiCD gene cluster sequence is 100% consistent. (TIF) [file pone.0196977.s002.tif]

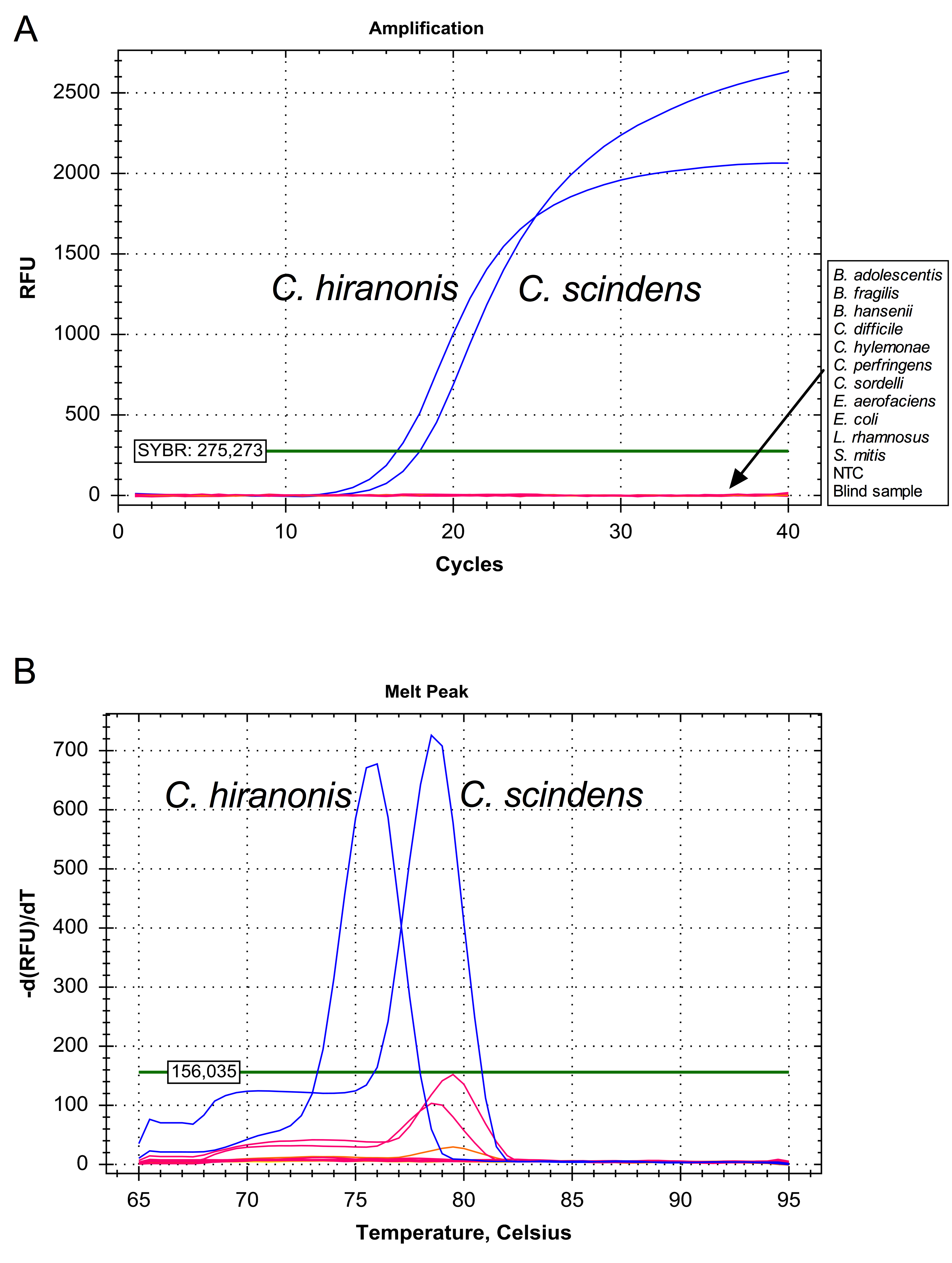

Supplement: S3 Fig — S3A Fig: Cross-reaction experiments with 10 ng DNA of different bacterial strains (red) demonstrated primer specificity for the baiCD genes of C. scindens (blue) and C. hiranonis (blue) with Ct values of 16.64 (C. scindens) and 18.01 (C. hiranonis), respectively. Yellow: Non-template control (NTC), orange: Blind sample, dark green: Automatically calculated threshold. S3B Fig: The corresponding melting curves for this experiment showed slightly different melting peaks for C. hiranonis and C. scindens. (TIFF) [file pone.0196977.s003.tiff]
